# Death from Ether

**Published:** 1874-02

**Authors:** 


Death from the Inhalation of Ether.—Our English brethren seem to have bad luck with anæsthetics. They are just beginning to appreciate the greater safety of ether as compared with chloro- orm, but a discouraging accident occurred, October 4th, at the South Hants Infirmary, Southampton. The patient, a lad of fourteen years, was put under the influence of ether for operation on the eye. Just before the operation, the pulse was felt to falter. The inhalation was discontinued, the pulse recovered, and the operation was performed without further inhalation. Directly after the operation, the pulse and respiration ceased simultaneously. Restorative means were resorted to, and at first with some success, but in a few minutes the boy was dead. The *post mortem* revealed nothing. The ether was administered by means of a cone. The quantity used is not stated, nor is any allusion made to its quality.

